# Efficacy and Safety of Inclisiran—An Assessment of the Consistency Between Randomized Controlled Trials and Real-World Evidence: A Systematic Review

**DOI:** 10.31083/RCM48272

**Published:** 2026-05-26

**Authors:** Yaru Lin, Haixiong Wang, Han Yang, Zekai Li

**Affiliations:** ^1^Academy of Medical Sciences, Shanxi Medical University, 030001 Taiyuan, Shanxi, China; ^2^Shanxi Key Laboratory of Heart Failure Precision Medicine, Cardiovascular Hospital Affiliated to Shanxi Medical University, 030024 Taiyuan, Shanxi, China; ^3^Department of Cardiology, The Second Hospital of Shanxi Medical University, 030001 Taiyuan, Shanxi, China

**Keywords:** inclisiran, cholesterol, LDL, randomized controlled trials as topic, real-world evidence, hypercholesterolemia, *PCSK9* inhibitors, diabetes mellitus, drug safety

## Abstract

**Background:**

This study aimed to compare the consistency in low-density lipoprotein cholesterol (LDL-C) reduction and the safety profile of the novel lipid-lowering agent inclisiran between randomized controlled trials (RCTs) and real-world evidence (RWE).

**Methods:**

A systematic search of the PubMed, Embase, and Web of Science databases (2022–2025) identified three RCTs (n = 1833) and five RWE studies (n = 805). LDL-C reduction in the RCTs at 540 days was compared with that observed at 90 days in the RWE studies. Subgroup analyses were performed for patients with and without diabetes.

**Results:**

Four of the five RWE studies showed complete overlap of confidence intervals with the RCT results. Weighted regression analysis demonstrated a strong positive association between the reduction in LDL-C reported in the RCTs and RWE studies (R^2^ = 0.774; *p* = 0.004). The diabetic subgroup exhibited significantly greater LDL-C reduction than the overall RWE population (*p* = 0.003), with absolute differences ranging from 2.3% to 4.8% compared with corresponding RCTs. Safety profiles were comparable across study types, with differences in adverse event incidence of less than 5%. Injection-site reactions were the most frequently reported adverse events.

**Conclusion:**

Inclisiran demonstrated highly consistent LDL-C-lowering efficacy and a comparable safety profile across both RCTs and real-world settings.

**The PROSPERO Registration:**

CRD42024618982, URL: https://www.crd.york.ac.uk/PROSPERO/view/CRD42024618982.

## 1. Introduction

Cardiovascular diseases (CVDs) constitute a major global public health challenge. According to the World Health Organization (WHO), CVDs remain the leading cause of death worldwide, with approximately 58% of CVD-related mortality occurring in Asia [1]. Among these conditions, atherosclerotic cardiovascular disease (ASCVD) and its major complications myocardial infarction (MI) and ischemic stroke are the predominant contributors to cardiovascular morbidity and mortality. Substantial evidence indicates that inadequate control of low-density lipoprotein cholesterol (LDL-C) plays a critical role in the development of ASCVD [2]. LDL-C is a well-established and independent risk factor for ASCVD [3]. Elevated LDL-C levels promote atherosclerotic plaque formation, leading to endothelial dysfunction and vascular inflammation, which ultimately precipitate adverse cardiovascular events such as MI and stroke [4]. This pathogenic process is particularly pronounced in individuals with diabetes. ASCVD is the leading cause of death and disability among patients with diabetes or prediabetes, who face a markedly increased risk of stroke, coronary heart disease, and myocardial infarction compared with individuals with normoglycemia [5]. In addition to its clinical impact, CVD imposes a substantial economic burden, with combined direct and indirect costs amounting to hundreds of billions of dollars annually. Consequently, effective preventive and therapeutic strategies are essential to mitigate the growing public health burden of cardiovascular disease. Statins are currently the cornerstone of LDL-C-lowering therapy in clinical practice [6]. However, their limited tolerability in some patients and the occurrence of adverse effects underscore the need for alternative lipid-lowering approaches. Inclisiran (Leqvio®) is a novel small interfering RNA (siRNA)-based therapy that targets proprotein convertase subtilisin/kexin type 9 (*PCSK9*). By inhibiting *PCSK9* expression through the endogenous RNA interference (RNAi) pathway, inclisiran effectively reduces circulating LDL-C levels [7, 8]. Beginning in 2017, the ORION series of randomized controlled trials (RCTs) established the clinical efficacy of inclisiran. These studies demonstrated that inclisiran achieved approximately a 50% reduction in LDL-C over a follow-up period of up to 540 days. Among them, the three pivotal Phase III trials ORION-9, ORION-10, and ORION-11 (hereafter collectively referred to as “RCT data”) provided the primary evidence supporting its clinical use [9]. In 2021, inclisiran received approval from the U.S. Food and Drug Administration for use in routine clinical practice. Since then, it has been formally adopted in several European countries, where accumulating real-world clinical data further support its effectiveness and safety. By 2024, approximately 500,000 doses of inclisiran had been administered in real-world settings [10]. RCTs are widely regarded as the gold standard for evaluating the efficacy and safety of novel therapeutic interventions. Under controlled conditions, they provide high-quality evidence that enables rigorous assessment of treatment effects. However, because RCTs typically enroll highly selected patient populations under strict inclusion and exclusion criteria, their findings may not fully reflect outcomes observed in routine clinical practice [11, 12]. RWE, derived from observational studies and everyday clinical care, complements RCT data by capturing treatment performance in broader and more heterogeneous patient populations. In addition, RWE offers valuable insights into medication adherence, long-term outcomes, and real-world cost-effectiveness. Consistency between RCTs and RWE is therefore crucial for validating the clinical utility of novel therapies and supporting evidence-based decision-making in practice [13]. In this review, we aim to evaluate the concordance of inclisiran’s LDL-C-lowering efficacy and safety profiles between RCTs and RWE, with the goal of informing clinical treatment strategies and therapeutic decision-making.

## 2. Materials and Methods

### 2.1 Search Strategy Inclusion Criteria, and Exclusion Criteria

We searched the PubMed, Embase, and Web of Science databases using the following keywords: (Inclisiran OR Leqvio) AND (“LDL Cholesterol” OR LDL-C) AND (“Randomized Controlled Trial” OR RCT OR “Real-World Evidence” OR “Real-World Data” OR “Observational Studies” OR Cohort). The search period spanned from January 1, 2022, to May 30, 2025, with the final search conducted on August 30, 2025. RCTs were restricted to Phase III trials or prospective/retrospective cohort studies with at least 50 participants. Eligible studies were required to report relative LDL-C reduction from baseline to the first assessment (90 days) or at 510 days, and to provide mean values with standard deviations, 95% confidence intervals, or equivalent measures. Ultimately, three RCTs (ORION-9, ORION-10, and ORION-11) and five real-world cohort studies were included. In addition to the efficacy-focused literature search, an alternative approach was used to identify key evidence for safety analysis. First, safety outcomes reported in the RWE studies identified in the initial search were assessed. Second, to incorporate the most comprehensive and up-to-date pharmacovigilance evidence from the U.S. Food and Drug Administration Adverse Event Reporting System (FAERS), a targeted search was conducted to identify large-scale post-marketing studies. The search terms “(Inclisiran OR Leqvio) AND (‘FAERS’ OR ‘Pharmacovigilance’ OR ‘Safety Profile’)” identified an important study by Li *et al*. (2025) [14], which provided a broad analysis of adverse event signals.

This systematic review was registered in PROSPERO (Registration Number: CRD42024618982). The literature search and selection process followed the PRISMA statement, as shown in **Supplementary Fig. 1**.

### 2.2 Study Selection and Data Extraction

Two reviewers (YL and HY) independently screened titles and abstracts, assessed full texts for eligibility, and extracted data using a standardized form. Any disagreements were resolved by consensus, with a third reviewer (HW) consulted if necessary.

### 2.3 Time Window and Consistency Determination Method

Phase III trials (ORION-9, ORION-10, and ORION-11) of inclisiran demonstrated that steady-state *PCSK9* inhibition was achieved within 30–60 days after the first dose, with LDL-C reduction fluctuations of less than 2% between 90 and 540 days [9]. Therefore, Day 90 was selected as the early real-world efficacy endpoint. Comparisons were conducted between the 90-day LDL-C reduction and the incidence of adverse events (AEs) in each real-world cohort and the corresponding 540-day 95% confidence interval (CI) from the RCTs [15]. If the observed reduction fell within this confidence interval, or if the absolute difference from the RCT point estimate was ≤5% [16], the result was defined as “consistent”.

### 2.4 Statistical Analysis

As this review involved qualitative comparisons without pooling effect sizes, no meta-analysis was performed. All data were directly extracted from the original studies. Overlap test: The proportion of CI overlap was calculated and defined as whether the CI of the RWE estimate included or intersected with the RCT point estimate. Weighted regression: A linear model adjusted for baseline LDL-C was constructed using inverse variance (1/SE^2^) as weights, with the formula as follows:

Yi = β0 + β1 × RWEi + β2 × BaselineLDLi + ϵiYi​ = β0​ + β1 × RWEi​ + β2 × BaselineLDLi​ + ϵi​

The weight wi = 1/SEi^2^. All statistical analyses were performed using R (Version: R 4.5.1 (2025-06-13); Manufacturer: R Core Team, The R Foundation for Statistical Computing; Location: Vienna, Austria.)

We did a descriptive summary of the safety outcomes from the included RCTs and RWE trials, as well as a more extensive study and comparison of safety utilizing the results of large-scale pharmacovigilance studies based on FAERS data.

### 2.5 Bias Risk Assessment

The risk of bias and methodological quality of all included studies were assessed using the Critical Appraisal Checklists of the Joanna Briggs Institute (JBI) [17, 18]. According to study design, the JBI RCT Checklist was applied to the three RCTs, while the JBI Cohort Study Checklist was used for the five RWE cohort studies. Two independent reviewers (YL and HY) conducted the assessments separately, and any disagreements were resolved through consultation with a third reviewer (HW).

Based on data extracted from the original studies, each appraisal item was rated as “Yes”, “No”, or “Unclear”. Overall study quality was determined by the total number of “Yes” ratings: high quality (≥7 “Yes” responses), moderate quality (5–6 “Yes” responses), and low quality (≤4 “Yes” responses). Detailed appraisal results are presented in Table 1 (Ref. [19, 20, 21, 22, 23, 24, 25]).

**Table 1.** **Risk of bias and quality assessment**.

Study	Random allocation/Clear research question	Allocation concealment/Minimize selection bias	Blinding/Reliable exposure measurement	Outcome blinding/Reliable outcome measurement	Baseline comparability/Address confounders	ITT analysis/Complete follow-up	Follow-up completeness/Appropriate statistical	Comparable co-interventions/Interpret limitations	Valid outcome measurement	Appropriate statistical	Overall bias risk/Quality
ORION-9 [19]	Yes	Yes	Yes	Yes	Yes	Yes	Yes	Yes	Yes	Yes	Low
ORION-10 [20]	Yes	Yes	Yes	Yes	Yes	Yes	Yes	Yes	Yes	Yes	Low
ORION-11 [20]	Yes	Yes	Yes	Yes	Yes	Yes	Yes	Yes	Yes	Yes	Low
Briani *et al*. (2025) [21]	Yes	Yes	Yes	Yes	Yes	Yes	Yes	Yes	-	-	High
Makhmudova *et al*. (2023) [22]	Yes	Yes	Yes	Yes	Yes	Yes	Yes	Yes	-	-	High
Padam *et al*. (2022) [23]	Yes	No	Yes	Yes	Yes	Unclear	Yes	Yes	-	-	Moderate
Mazdeyasnan *et al*. (2025) [24]	Yes	Yes	Yes	Yes	Yes	Yes	Yes	Yes	-	-	High
Iqbal *et al*. (2024) [25]	Yes	No	Yes	Yes	No	Yes	Yes	Yes	-	-	Moderate

Abbreviation: ITT, Intention-To-Treat.

As shown in Table 1, all three RCTs were assessed as having a low risk of bias, indicating that their study design and implementation met the methodological standards of high-quality randomized trials and that potential bias was unlikely to substantially affect the validity of the results. Among the five RWE cohort studies, three were rated as high quality and two as moderate quality. The primary methodological limitations of the moderate-quality studies were their single-center design, which may have introduced selection bias, and insufficient adjustment for confounding factors. Overall, the included studies demonstrated a low risk of bias, and the identified methodological limitations were unlikely to materially affect the internal validity of the findings.

## 3. Result

### 3.1 Baseline Data

Analysis of baseline data (Table 2A,2B, Ref. [19, 20, 21, 22, 23, 24, 25]) showed no significant difference in baseline LDL-C levels between the RWE and RCT cohorts (*p* = 0.28), indicating that the study populations were comparable. Regarding the primary efficacy endpoint, LDL-C reductions observed in RWE were highly consistent with those reported in RCTs, with no statistically significant difference (*p* = 0.75). Notably, subgroup analysis in patients with diabetes revealed a significantly greater LDL-C reduction compared with the overall study population (*p* = 0.003), suggesting a potentially enhanced efficacy of inclisiran in this subgroup. The baseline characteristics of participants included in this analysis were consistent with those reported in the ORION series trials and the CHOLINET Registry study. Detailed stratification data are available in the publicly accessible datasets of the original studies (ORION series: [15]; CHOLINET: [15]).

**Table 2A.** **Summary of LDL-C reduction in RCTs and RWE**.

Study	Type	Region	Population	n	Baseline LDL-C, mmol/L, mean (SD)	LDL-C reduction mmol/L, mean (SD)	LDL-C reduction% (95% CI)	Corresponding RCT
ORION-9 [19]	RCT	European	HeFH	242	3.92 (1.27)	1.50 (0.98)	39.7 (35.7∼43.7)	___
		South Africa						
ORION-10 [20]	RCT	USA	ASCVD	781	2.70 (1.02)	1.41 (1.01)	51.3 (48.8∼55.7)	___
ORION-11 [20]	RCT	European	ASCVD	810	3.70 (1.70)	1.32 (0.99)	49.8 (46.6∼53.1)	___
		South Africa						
Briani *et al*. (2025) [21]	RWE	Italy	ASCVD/HeFH	240	3.07 (1.26)	1.47 (1.04)	52.3 (45.6∼59.44)	ORION-11
Makhmudova *et al*. (2023) [22]	RWE	Germany	ASCVD	153	3.6 (1.2)	1.9 (1.12)	41.1 (35.5∼46.7)	ORION-11
Padam *et al*. (2022) [23]	RWE	UK	ASCVD	80	3.5 (1.2)	1.8 (1.0)	48.6 (42.6∼54.6)	ORION-10
Mazdeyasnan *et al*. (2025) [24]	RWE	USA	ASCVD	186	2.77 (1.22)	1.37 (0.75)	48 (42.4∼54.1)	ORION-10
Iqbal *et al*. (2024) [25]	RWE	Middle East	ASCVD/HeFH	146	3.0 (1.33)	1.8 (0.90)	47.7 (41.6∼53.8)	ORION-9

Abbreviation: LDL-C, low-density lipoprotein cholesterol; RCTs, randomized controlled trials; RWE, real-world evidence; CI, confidence interval; HeFH, Heterozygous Familial Hypercholesterolemia; ASCVD, atherosclerotic cardiovascular disease.

**Table 2B.** **Summary and comparative analysis of LDL-C reduction in RCTs and RWE across diabetes subgroups**.

Study	Type	Region	n	Baseline LDL-C, mmol/L, mean (SD)	LDL-C reduction% (95% CI)	Diabetes subgroup LDL-C reduction% (95% CI)	Corresponding RCT diabetes subgroup
ORION-9 [19]	RCT	European	58	3.99 (1.57)	39.7 (35.7∼43.7)	51.9 (55.7∼48.1)	/
		South Africa					
ORION-10 [20]	RCT	USA	371	2.90 (1.42)	51.3 (48.8∼55.7)	55.2 (60.9∼49.9)	/
ORION-11 [21]	RCT	European	296	3.75 (1.76)	49.8 (46.6∼53.1)	56.3 (61.6∼51.5)	/
		South Africa					
Briani *et al*. (2025) [21]	RWE	Italy	60	3.13 (1.13)	52.3 (45.6∼59.4)	59.9 (70.9∼47.9)	ORION-11
Makhmudova *et al*. (2023) [22]	RWE	Germany	30	3.60 (1.20)	41.1 (35.3∼46.7)	48.1 (53.7∼42.5)	ORION-11
Padam *et al*. (2022) [23]	RWE	UK	19	3.50 (1.20)	48.6 (42.6∼54.6)	50.6 (56.6∼44.6)	ORION-10
Mazdeyasnan *et al*. (2025) [24]	RWE	USA	56	2.77 (1.22)	48.0 (42.4∼54.1)	50.3 (56.1∼44.4)	ORION-10
Iqbal *et al*. (2024) [25]	RWE	Middle East	89	3.00 (1.33)	47.7 (41.6∼53.8)	49.7 (55.8∼43.6)	ORION-9

### 3.2 RCT and RWE Consistency Analysis

**Fig. 1.**
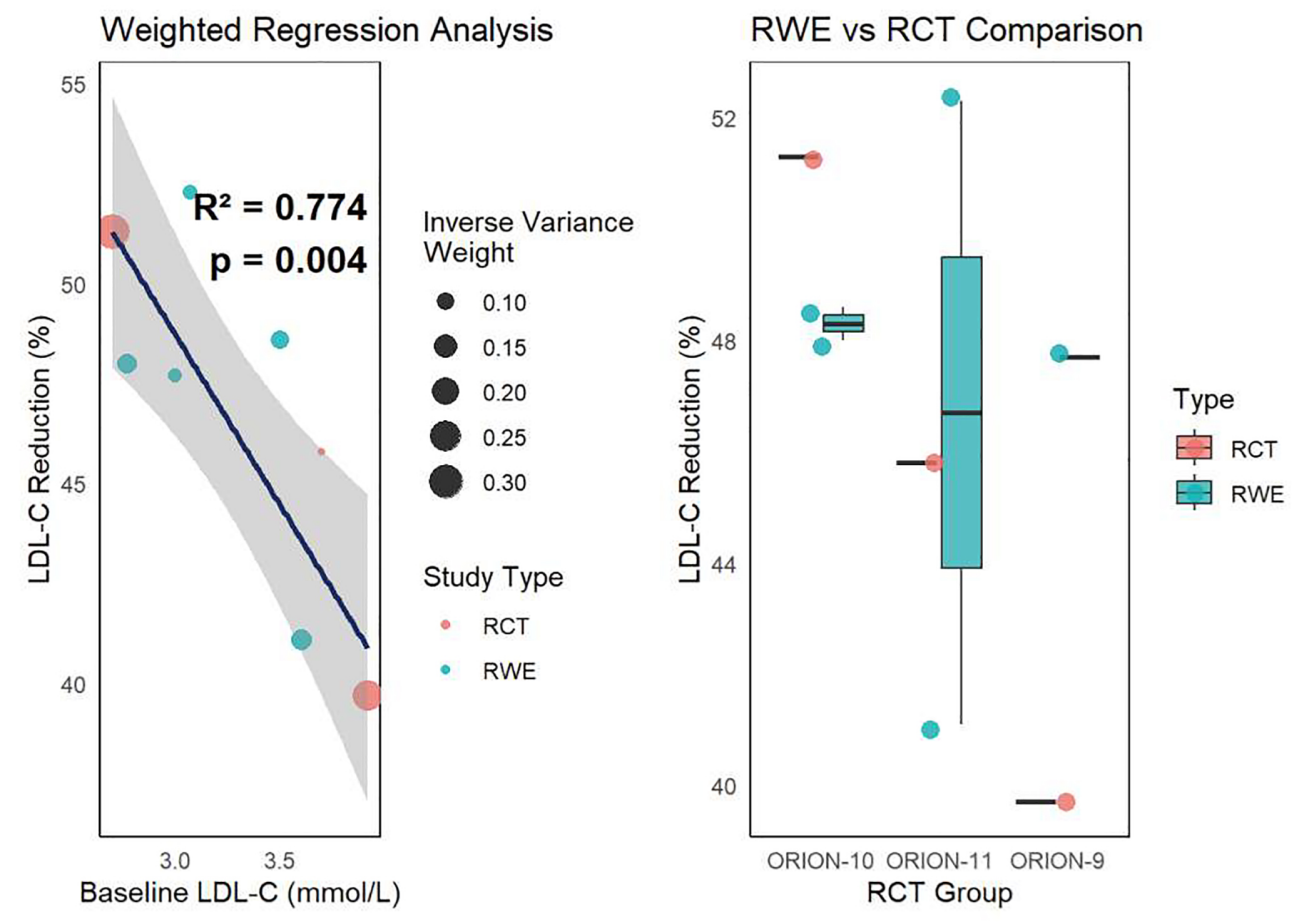
**Weighted regression analysis of LDL-C reduction in RWE studies and RCTs**. CI, confidence interval; LDL-C, low-density lipoprotein cholesterol; RCTs, randomized controlled trials; RWE, real-world evidence.

Overlap analysis (Table 3, Ref. [21, 22, 23, 24, 25]) showed that, among the five comparisons, four RWE studies exhibited complete overlap with the corresponding RCT point estimates, while one showed partial overlap. No instance of complete non-overlap was observed, indicating a high degree of statistical consistency between RCT and RWE results. Weighted regression analysis (Fig. 1) demonstrated a significant positive correlation between LDL-C reduction in RCTs and RWE studies (R^2^ = 0.774, *p* = 0.004), suggesting that RCT data can reliably predict real-world outcomes. The regression model indicated that neither baseline LDL-C levels nor study type (RCT vs RWE) significantly influenced outcome variability, further supporting the consistency between the two types of studies.

**Table 3.** **Overlap analysis of 95% confidence intervals for LDL-C reduction between RCTs 540 days and RWE 90 days**.

RWE_Study	RCT_Study	RWE _Point_Estimate	RWE_CI_Lower	RWE_CI_Upper	RCT_Point_Estimate	Overlap_Type
Briani *et al*. (2025) [21]	ORION-11	52.3	45.6	59.4	45.8	Full overlap
Makhmudova *et al*. (2023) [22]	ORION-11	41.1	35.5	46.7	45.8	Full overlap
Padam *et al*. (2022) [23]	ORION-10	48.6	42.6	54.6	51.3	Full overlap
Mazdeyasnan *et al*. (2025) [24]	ORION-10	48.0	42.4	54.1	51.3	Full overlap
Iqbal *et al*. (2024) [25]	ORION-9	47.7	41.6	55.8	39.7	Partial overlap
Overall summary		NA	NA	NA	NA	Full overlap: 4
						Partial overlap: 1
						No overlap: 0

NA: not applicable.

### 3.3 Comparative Analysis of LDL-C Reduction Between RWE and RCTs in the Diabetes Subgroup

**Fig. 2.**
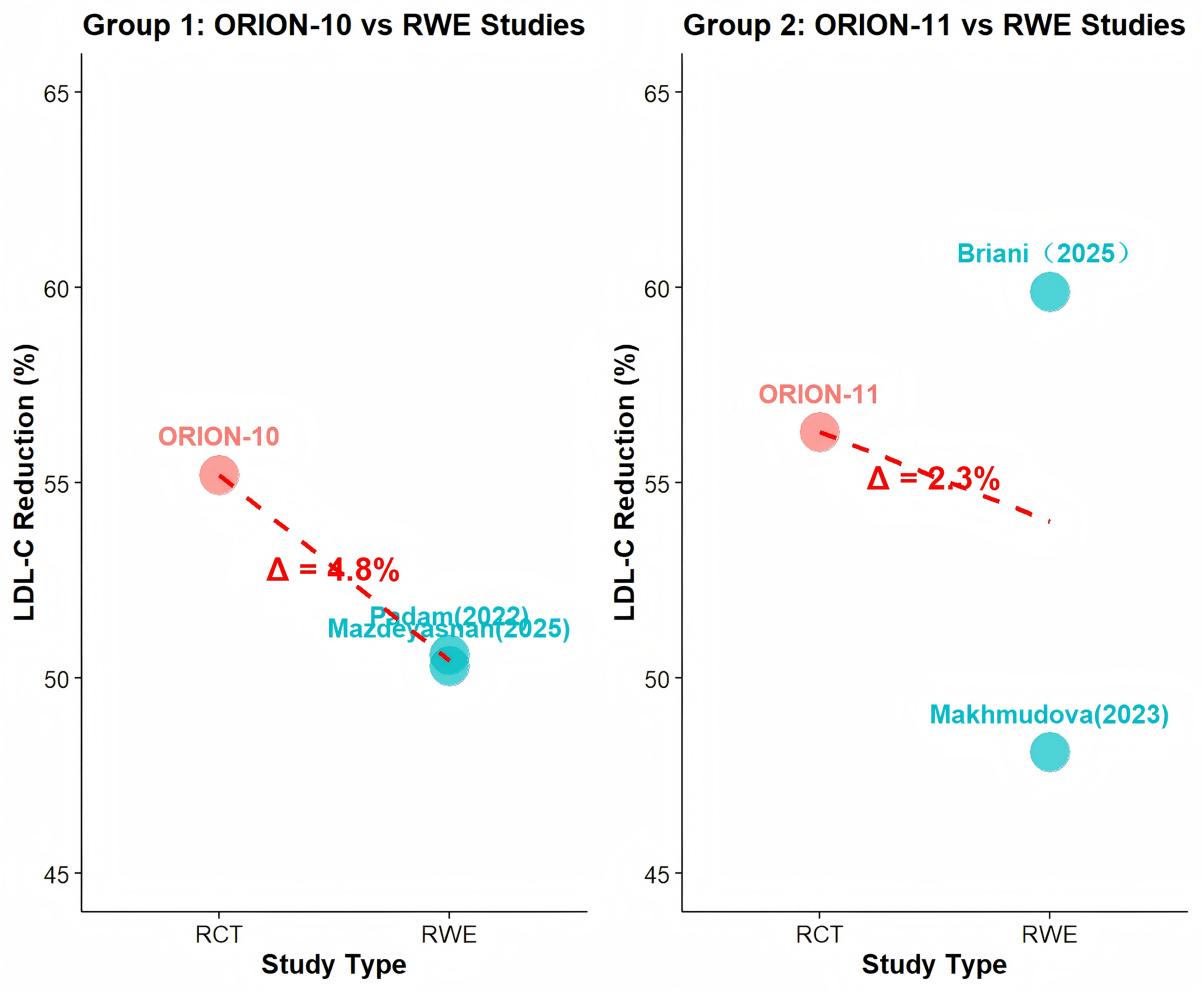
**Efficacy and consistency analysis of LDL-C reduction in the diabetes subgroup for RWE and RCTs**.

For the diabetes subgroup analysis, because no corresponding RWE cohort was available in ORION-9, ORION-10 and ORION-11 were divided into two groups to assess consistency between RWE and RCTs (Fig. 2). In Group 1, the percentage difference in LDL-C reduction between RCTs and RWE was 4.8%, while in Group 2 it was 2.3%, both below 5%. These results indicate that LDL-C reduction is highly consistent within the diabetes subgroup.

### 3.4 Summary of Safety Evidence for Inclisiran

The safety profile of inclisiran (Table 4, Ref. [14, 21, 22, 23, 24, 25, 26]) was consistent across all included RWE studies, with differences of less than 5% compared to the safety profile reported in randomized controlled trials. Mild to moderate injection-site reactions were the most frequently reported adverse events. Data from the FAERS database provide further confirmation, identifying injection-site discomfort as the most prominent disproportionality signal (Reporting Odds Ratio [ROR] = 11.87). Notable signals were also observed for established musculoskeletal events, including arthralgia (ROR = 5.45) and myalgia (ROR = 14.97). These findings support the favorable benefit-risk profile of inclisiran observed in clinical trials.

**Table 4.** **Summary of safety evidence for inclisiran**.

Study	Type	Most common adverse event(s)	Incidence%**/**Signal strength (ROR 95% CI)	Notes & Key findings
Koenig W *et al*. (2024) (ORION-9/10/11) [26]	RCT	Injection site reaction	3.1	Established safety benchmark.
				Most AEs were mild to moderate and self-limiting.
Briani *et al*. (2025) [21]	RWE	Injection site reaction	1.3	Consistent with RCTs.
Makhmudova *et al*. (2023) [22]	RWE	Injection site reaction	2.6	Consistent with RCTs.
Padam *et al*. (2022) [23]	RWE	Injection site reaction	1.3	Consistent with RCTs.
Mazdeyasnan *et al*. (2025) [24]	RWE	Injection site reaction	1.7	Consistent with RCTs.
Iqbal *et al*. (2024) [25]	RWE	Injection site reaction	0.1	Consistent with RCTs.
Pharmacovigilance	FAERS	Injection site pain	ROR = 11.87 (10.69, 13.18)	Strongest signals validate known risks
Li B *et al*. (2025) [14]		Arthralgia	ROR = 5.45 (4.88, 6.08)	Identified potential new signals (e.g., movement disorder)
(*Expert Opinion on Drug Safety*)		Myalgia	ROR = 14.97 (13.19, 16.99)	(See text for details on serious outcomes*)

Abbreviation: AEs, adverse events; FAERS, U.S. Food and Drug Administration Adverse Event Reporting System; ROR, Reporting Odds Ratio. *The FAERS analysis also reported cases with serious outcomes, including 58 fatalities and 132 hospitalizations. It is important to note that these events have not been confirmed as causally related to inclisiran. Given that the patient population receiving inclisiran is inherently at high risk for ASCVD, these serious outcomes are most likely attributable to the natural progression of the underlying disease rather than to the medication.

## 4. Discussion

This review, based on a qualitative analysis of RCTs and RWE, demonstrates that inclisiran has highly consistent efficacy in lowering LDL-C across both study types. Integrated analysis of RCTs and RWE further confirms that inclisiran achieves stable and durable LDL-C-lowering effects in high-risk patients with hypercholesterolemia, with efficacy consistently observed across different age groups and comorbidity subgroups. Notably, all patients included in this study received stable-dose statin therapy, with some patients co-administered ezetimibe. The potential influence of concomitant lipid-lowering drugs on efficacy was further assessed through evidence-based literature: the effect of concomitant lipid-lowering therapy on treatment outcomes has been supported by evidence-based studies [26] (CHOLINET: https://clinicaltrials.gov/study/NCT03399370), which did not significantly affect the main conclusions. This finding aligns with the established mechanism of action of inclisiran lowers LDL-C by specifically inhibiting *PCSK9* synthesis, and its efficacy is not substantially influenced by baseline statin monotherapy or combination therapy with ezetimibe. Among the five included RWE studies, four showed complete overlap in confidence intervals with their corresponding RCTs, while one showed only partial overlap. This variation is within an acceptable range and may be attributed to higher medication adherence in RCT populations compared with RWE settings, likely due to closer monitoring, more frequent follow-ups, and higher baseline adherence levels [27, 28]. Furthermore, weighted regression analysis demonstrated a significant positive correlation between LDL-C reduction rates in RWE and RCTs, indicating that RCT data can reliably predict real-world outcomes and further supporting the consistency between the two types of studies. This finding shows that inclisiran not only performs effectively in strictly controlled clinical trial settings but also maintains stable efficacy in real-world clinical practice. Compared with patients enrolled in the ORION study protocols, inclisiran is applied to a broader population in real-world settings, including patients with statin intolerance and those receiving concomitant therapy with ezetimibe, fenofibrate, or lipoprotein apheresis [22]. These observations provide strong support for the broader clinical application of inclisiran in daily practice. Diabetic patients are at high risk of cardiovascular disease; therefore, controlling LDL-C levels in this population is crucial for the prevention of cardiovascular events. Subgroup analysis in diabetic patients revealed that inclisiran significantly reduced LDL-C levels compared with the overall study population. Consistency analyses further showed that the efficacy difference between RWE and RCTs was within 5%, indicating good agreement. These results suggest that inclisiran may have a more favorable efficacy profile in diabetic patients, offering a new therapeutic option for cholesterol management, particularly when standard statin therapy is ineffective or poorly tolerated. However, given the small sample size of this subgroup, these findings require validation in larger-scale studies.

In terms of safety, existing data indicate that the incidence of AEs associated with inclisiran shows a generally consistent pattern between RCTs and RWE. Injection-site reactions are the most common AEs, which are mostly mild and reversible [29], and this finding is largely consistent with the safety profile reported in RCTs. Additionally, our analysis of the latest pharmacovigilance data from FAERS [14] not only confirms injection-site reactions as the primary safety signal but also quantifies and highlights the occurrence of musculoskeletal events, including myalgia and arthralgia. This underscores the importance of proactively identifying and interpreting potential causal relationships among emerging adverse reactions in real-world clinical settings. However, the safety analysis in this study has several limitations. First, only one of the included RWE studies considered safety as the primary endpoint, resulting in relatively limited data support. Second, FAERS data have inherent limitations, including susceptibility to reporting bias, duplicate records, and the absence of denominators for exposed populations, which introduces uncertainty in data interpretation. Therefore, caution should be exercised regarding the current conclusion of “consistent safety”, and overinterpretation should be avoided. Existing evidence can only tentatively suggest that the benefit-risk profile of inclisiran is generally manageable, while its long-term safety requires verification in more targeted studies. At the same time, a potential limitation of inclisiran should be acknowledged: due to its long-acting effect of up to six months, any adverse reactions occurring after long-term treatment may be difficult to reverse [26, 30]. This further emphasizes the need for long-term follow-up studies with safety as the primary endpoint.

The evidence base of this study also has limitations. The included RWE dataset is relatively small, comprising five cohorts with a total of 805 patients, and most study populations are from high-income regions, including Italy, Germany, the UK, the US, and the Middle East. Data from low- and middle-income regions and from broader ethnic or racial populations are lacking, which may limit the external validity and generalizability of the findings [31, 32]. Although RWE has, to some extent, expanded the extrapolation of traditional RCTs and addressed certain limitations of clinical trials, practical factors in real-world settings such as variations in patient adherence, treatment cost burdens, medical insurance coverage, and complex comorbidities may still influence the final outcomes of cholesterol management. Even in the era of *PCSK9* inhibitors, the rate of achieving lipid-control targets in clinical practice remains suboptimal [33]. These observations suggest that future studies should expand sample sizes, include more diverse regions and populations, and conduct multicenter, long-term follow-up RWE studies. Such efforts would not only validate the efficacy and safety of inclisiran in broader populations but also help identify effective strategies to optimize lipid management in clinical practice, thereby improving the rate of achieving lipid-control targets.

### Limitations

Diabetic subgroup constraints: Small RWE samples, wide confidence intervals, and 2–4% LDL-C reduction near measurement noise. Unadjusted for baseline LDL-C/concomitant therapies, this ecological comparison cannot support “better effectiveness” in diabetics.

Inadequate safety analysis: Only one RWE study focused on safety; FAERS data flaws (bias, duplicates, no denominators) cause interpretive uncertainty. Caution is needed to avoid over-optimism about safety consistency.

Narrow evidence base: Modest RWE dataset from high-income regions only, limiting external validity and generalizability.

## 5. Conclusion

In conclusion, this study, based on integrated RCT and RWE data, confirms that inclisiran demonstrates highly consistent efficacy in lowering LDL-C among high-risk patients with hypercholesterolemia. This efficacy is not significantly affected by baseline statin monotherapy or ezetimibe combination therapy, providing evidence-based support for its clinical use. Subgroup analysis indicates that inclisiran also achieves stable LDL-C reduction in patients with diabetes complicated by hypercholesterolemia, offering an alternative option for those with statin intolerance or poor response. However, the analysis focused solely on LDL-C-lowering efficacy as a single endpoint, and the limited sample size restricts a comprehensive evaluation of inclisiran’s overall therapeutic benefits. Additionally, a formal risk of bias assessment has been conducted, but the sample size was small, which may affect the internal validity of the results. Future research should expand sample sizes, extend follow-up periods, include populations from low- and middle-income regions and diverse ethnic groups, and incorporate risk assessments and cost-effectiveness analyses to verify the long-term efficacy and safety of inclisiran across diverse populations.

## Availability of Data and Materials

Data sharing not applicable to this article as no datasets were generated or analyzed during the current study.
