# Supplementary material for: Efficacy and Safety of Inclisiran—An Assessment of the Consistency Between Randomized Controlled Trials and Real-World Evidence: A Systematic Review
Source: Rev Cardiovasc Med. 2026 May 26;27(5):48272. doi: 10.31083/RCM48272 (PMC13227393; doi:10.31083/RCM48272)
Supplement: Supplementary file 1 [file 2153-8174-27-5-48272-s1.zip › Supplementary Material.docx]

**Supplementary Fig. 1 PRISMA Flow Diagram: Literature Search and Selection Process**

**Identification of studies via databases**

Records removed *before screening*:

Duplicate records removed (n = 10)

Records marked as ineligible by automation tools (n = 13)

Records identified from*:

Databases (n = 291)

**Identification**

Records screened

(n =268 )

Records excluded**

(n = 23)

Reports sought for retrieval

(n =245 )

Reports not retrieved

(n = 218)

**Screening**

Reports assessed for eligibility

(n = 19)

Reports excluded:

Reason 1.Sample size<50 (n = 4)

Reason 2.Lack of key data )

(n =7 )

Studies included in RCTs

(n =3 )

RWE (n =5 )

**Included**
